# Poly[bis­(μ-hemihydrogen 2-phenyl­quinoline-4-carboxyl­ato-κ^2^ *N*,*O*)silver(I)]

**DOI:** 10.1107/S1600536809001184

**Published:** 2009-01-23

**Authors:** Xiutang Zhang, Peihai Wei, Bin Li

**Affiliations:** a Advanced Material Institute of Research, Department of Chemistry and Chemical Engineering, Shandong Institute of Education, Jinan, 250013, People’s Republic of China; b College of Chemistry and Chemical Engineering, Liaocheng University, Liaocheng, 252059, People’s Republic of China; c Department of Chemistry and Chemical Engineering, Shandong Institute of Education, Jinan 250013, People’s Republic of China

## Abstract

In the title compound, [Ag(C_16_H_10.5_NO_2_)_2_], the Ag^I^ cation (site symmetry 2) is coordinated by two N atoms in a near-linear AgN_2_ arrangement. Two carboxyl­ate O atoms from two additional 2-phenyl­quinoline-4-carboxyl­ate ligands form long Ag—O bonds [2.6585 (17) Å], resulting in a distorted square-planar arrangement. The bridging ligands result in infinite corrugated sheets propagating in (010). An O—H⋯O hydrogen bond, disordered about a twofold axis, completes the structure.

## Related literature

For the related coordination polymers containing Mn^II^, Co^II^ and Cu^II^, see: Xiao *et al.* (2005 ▶); Xie *et al.* (2005 ▶) and Xie *et al.* (2006 ▶), respectively.
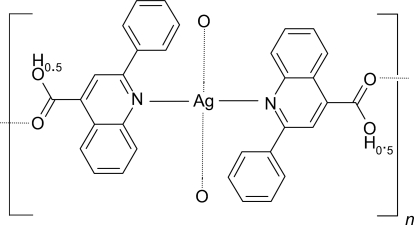


## Experimental

### 

#### Crystal data

[Ag(C_16_H_10.5_NO_2_)_2_]
*M* *_r_* = 605.38
Monoclinic, 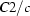

*a* = 7.2163 (6) Å
*b* = 20.5060 (17) Å
*c* = 16.5632 (12) Å
β = 97.585 (3)°
*V* = 2429.5 (3) Å^3^
*Z* = 4
Mo *K*α radiation
μ = 0.87 mm^−1^
*T* = 293 (2) K
0.20 × 0.15 × 0.15 mm

#### Data collection

Bruker SMART CCD diffractometer
Absorption correction: multi-scan (*SADABS*; Bruker, 2001 ▶) *T*_min_ = 0.877, *T*_max_ = 1.00 (expected range = 0.769–0.877)
9257 measured reflections
2791 independent reflections
2550 reflections with *I* > 2σ(*I*)
*R*_int_ = 0.020

#### Refinement

*R*[*F* ^2^ > 2σ(*F* ^2^)] = 0.029
*wR*(*F* ^2^) = 0.067
*S* = 1.10
2791 reflections
177 parameters
H-atom parameters constrained
Δρ_max_ = 0.66 e Å^−3^
Δρ_min_ = −0.33 e Å^−3^

### 

Data collection: *SMART* (Bruker, 2001 ▶); cell refinement: *SAINT* (Bruker, 2001 ▶); data reduction: *SAINT*; program(s) used to solve structure: *SHELXS97* (Sheldrick, 2008 ▶); program(s) used to refine structure: *SHELXL97* (Sheldrick, 2008 ▶); molecular graphics: *SHELXTL* (Sheldrick, 2008 ▶); software used to prepare material for publication: *SHELXTL*.

## Supplementary Material

Crystal structure: contains datablocks global, I. DOI: 10.1107/S1600536809001184/hb2825sup1.cif

Structure factors: contains datablocks I. DOI: 10.1107/S1600536809001184/hb2825Isup2.hkl

Additional supplementary materials:  crystallographic information; 3D view; checkCIF report

## Figures and Tables

**Table:** 

Ag1—N1	2.2413 (15)

**Table:** 

N1—Ag1—N1^i^	177.38 (8)

Symmetry code: (i) 
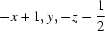
.

**Table 2:** Hydrogen-bond geometry (Å, °)

*D*—H⋯*A*	*D*—H	H⋯*A*	*D*⋯*A*	*D*—H⋯*A*
O1—H1O⋯O1^ii^	0.82	1.68	2.470 (3)	162

Symmetry code: (ii) 
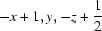
.

**Fig. 1.**
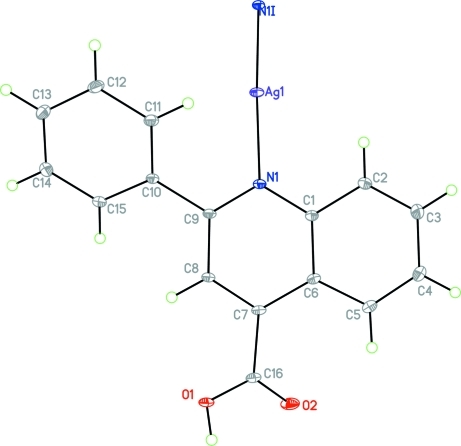
A view of the structure of (I), expanded to show the Ag—N bonds, showing 30% probability displacement ellipsoids. Symmetry code: I: 1–x, y, –z–1/2.

**Fig. 2.**
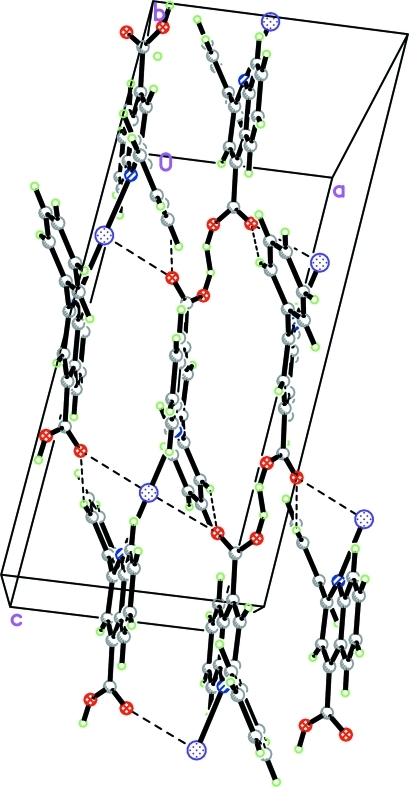
Part of a polymeric sheet in (I).

## supplementary crystallographic information

### Experimental

A mixture of silver nitrate (0.5 mmol) and 2-phenyl-4-quinolinecarboxylic acid (0.5 mmol) in H_2_O (8 ml) and ethanol (8 ml) was sealed in a 25 ml Teflon-lined stainless steel autoclave and kept at 413 K for three days.  Orange prisms of (I) were obtained after cooling to room temperature with a yield of 27%. Anal. Calc. for C_32_H_21_AgN_2_O_4_: C 63.43, H 3.47, N 4.63%; Found: C 63.41, H 3.32, N 4.59%.

### Refinement

The H atoms were placed in calculated positions (C—H = 0.93 Å, O—H = 0.82Å) and refined as riding with and *U*_iso_(H) = 1.2*U*_eq_(carruer).

### Crystal data

**Table:**

[Ag(C_16_H_10.5_NO_2_)_2_]	*F*(000) = 1224
*M**_r_* = 605.38	*D*_x_ = 1.655 Mg m^−^^3^
Monoclinic, *C*2/*c*	Mo *K*α radiation, λ = 0.71073 Å
Hall symbol: -C 2yc	Cell parameters from 3290 reflections
*a* = 7.2163 (6) Å	θ = 2.3–27.5°
*b* = 20.5060 (17) Å	µ = 0.87 mm^−^^1^
*c* = 16.5632 (12) Å	*T* = 293 K
β = 97.585 (3)°	Prism, orange
*V* = 2429.5 (3) Å^3^	0.20 × 0.15 × 0.15 mm
*Z* = 4	

### Data collection

**Table:**

Bruker SMART CCD diffractometer	2791 independent reflections
Radiation source: fine-focus sealed tube	2550 reflections with *I* > 2σ(*I*)
graphite	*R*_int_ = 0.020
ω scans	θ_max_ = 27.5°, θ_min_ = 2.3°
Absorption correction: multi-scan (*SADABS*; Bruker, 2001)	*h* = −9→9
*T*_min_ = 0.877, *T*_max_ = 1.00	*k* = −25→26
9257 measured reflections	*l* = −21→20

### Refinement

**Table:**

Refinement on *F*^2^	Primary atom site location: structure-invariant direct methods
Least-squares matrix: full	Secondary atom site location: difference Fourier map
*R*[*F*^2^ > 2σ(*F*^2^)] = 0.029	Hydrogen site location: inferred from neighbouring sites
*wR*(*F*^2^) = 0.067	H-atom parameters constrained
*S* = 1.10	*w* = 1/[σ^2^(*F*_o_^2^) + (0.0323*P*)^2^ + 1.551*P*] where *P* = (*F*_o_^2^ + 2*F*_c_^2^)/3
2791 reflections	(Δ/σ)_max_ < 0.001
177 parameters	Δρ_max_ = 0.66 e Å^−^^3^
0 restraints	Δρ_min_ = −0.33 e Å^−^^3^

### Special details

**Table:**

Geometry. All e.s.d.'s (except the e.s.d. in the dihedral angle between two l.s. planes) are estimated using the full covariance matrix. The cell e.s.d.'s are taken into account individually in the estimation of e.s.d.'s in distances, angles and torsion angles; correlations between e.s.d.'s in cell parameters are only used when they are defined by crystal symmetry. An approximate (isotropic) treatment of cell e.s.d.'s is used for estimating e.s.d.'s involving l.s. planes.
Refinement. Refinement of *F*^2^ against ALL reflections. The weighted *R*-factor *wR* and goodness of fit *S* are based on *F*^2^, conventional *R*-factors *R* are based on *F*, with *F* set to zero for negative *F*^2^. The threshold expression of *F*^2^ > σ(*F*^2^) is used only for calculating *R*-factors(gt) *etc*. and is not relevant to the choice of reflections for refinement. *R*-factors based on *F*^2^ are statistically about twice as large as those based on *F*, and *R*- factors based on ALL data will be even larger.

### Fractional atomic coordinates and isotropic or equivalent isotropic displacement parameters (Å^2^)

**Table:**

	*x*	*y*	*z*	*U*_iso_*/*U*_eq_	Occ. (<1)
Ag1	0.5000	0.224313 (10)	−0.2500	0.03976 (9)	
N1	0.4524 (2)	0.22681 (7)	−0.11903 (9)	0.0305 (3)	
O1	0.4807 (3)	0.16931 (8)	0.17478 (8)	0.0506 (4)	
H1O	0.4750	0.1750	0.2234	0.061*	0.50
O2	0.6459 (3)	0.26118 (9)	0.18437 (10)	0.0555 (4)	
C1	0.4841 (3)	0.28414 (9)	−0.07613 (11)	0.0323 (4)	
C2	0.4827 (4)	0.34294 (10)	−0.12074 (13)	0.0468 (5)	
H2A	0.4619	0.3417	−0.1773	0.056*	
C3	0.5113 (4)	0.40127 (11)	−0.08202 (16)	0.0562 (7)	
H3A	0.5094	0.4396	−0.1121	0.067*	
C4	0.5437 (4)	0.40349 (11)	0.00334 (16)	0.0546 (6)	
H4A	0.5627	0.4436	0.0294	0.065*	
C5	0.5477 (3)	0.34808 (11)	0.04857 (13)	0.0446 (5)	
H5A	0.5712	0.3506	0.1051	0.054*	
C6	0.5165 (3)	0.28627 (9)	0.01036 (11)	0.0317 (4)	
C7	0.5077 (3)	0.22584 (9)	0.05293 (11)	0.0313 (4)	
C8	0.4621 (3)	0.17105 (10)	0.00812 (11)	0.0344 (4)	
H8A	0.4467	0.1319	0.0348	0.041*	
C9	0.4376 (3)	0.17227 (9)	−0.07805 (10)	0.0302 (4)	
C10	0.3928 (3)	0.11103 (9)	−0.12467 (11)	0.0331 (4)	
C11	0.2740 (3)	0.11181 (10)	−0.19829 (12)	0.0379 (4)	
H11A	0.2219	0.1510	−0.2185	0.045*	
C12	0.2330 (3)	0.05444 (12)	−0.24147 (13)	0.0476 (5)	
H12A	0.1558	0.0555	−0.2911	0.057*	
C13	0.3063 (4)	−0.00390 (12)	−0.21119 (15)	0.0570 (7)	
H13A	0.2773	−0.0424	−0.2399	0.068*	
C14	0.4230 (4)	−0.00520 (11)	−0.13802 (15)	0.0593 (7)	
H14A	0.4731	−0.0446	−0.1177	0.071*	
C15	0.4659 (4)	0.05159 (11)	−0.09489 (12)	0.0463 (5)	
H15A	0.5442	0.0502	−0.0456	0.056*	
C16	0.5497 (3)	0.22006 (10)	0.14510 (11)	0.0356 (4)	

### Atomic displacement parameters (Å^2^)

**Table:**

	*U*^11^	*U*^22^	*U*^33^	*U*^12^	*U*^13^	*U*^23^
Ag1	0.05874 (17)	0.04506 (14)	0.01656 (11)	0.000	0.00894 (9)	0.000
N1	0.0388 (9)	0.0352 (8)	0.0176 (7)	0.0017 (7)	0.0040 (6)	0.0002 (6)
O1	0.0877 (12)	0.0478 (8)	0.0152 (6)	−0.0092 (8)	0.0029 (7)	−0.0008 (6)
O2	0.0607 (11)	0.0753 (11)	0.0275 (8)	−0.0222 (9)	−0.0048 (7)	−0.0068 (7)
C1	0.0384 (10)	0.0364 (10)	0.0232 (9)	0.0005 (8)	0.0081 (8)	−0.0004 (7)
C2	0.0718 (16)	0.0412 (11)	0.0289 (10)	0.0025 (11)	0.0122 (10)	0.0043 (8)
C3	0.085 (2)	0.0358 (11)	0.0494 (14)	−0.0001 (11)	0.0162 (13)	0.0038 (10)
C4	0.0766 (18)	0.0373 (11)	0.0498 (14)	−0.0021 (11)	0.0084 (12)	−0.0110 (10)
C5	0.0549 (14)	0.0462 (12)	0.0330 (11)	0.0004 (10)	0.0066 (10)	−0.0110 (9)
C6	0.0331 (10)	0.0391 (10)	0.0236 (9)	0.0001 (8)	0.0060 (7)	−0.0047 (7)
C7	0.0313 (10)	0.0447 (10)	0.0179 (8)	0.0001 (8)	0.0036 (7)	−0.0016 (7)
C8	0.0485 (12)	0.0369 (9)	0.0179 (8)	−0.0024 (9)	0.0046 (8)	0.0019 (7)
C9	0.0364 (10)	0.0368 (9)	0.0176 (8)	0.0005 (8)	0.0039 (7)	0.0010 (7)
C10	0.0457 (12)	0.0347 (9)	0.0194 (8)	−0.0039 (8)	0.0066 (8)	0.0005 (7)
C11	0.0447 (12)	0.0434 (11)	0.0252 (9)	−0.0031 (9)	0.0035 (8)	0.0000 (8)
C12	0.0557 (14)	0.0549 (13)	0.0309 (10)	−0.0146 (11)	0.0007 (10)	−0.0074 (9)
C13	0.088 (2)	0.0440 (12)	0.0401 (12)	−0.0171 (12)	0.0113 (12)	−0.0106 (10)
C14	0.101 (2)	0.0359 (11)	0.0414 (13)	0.0018 (12)	0.0110 (13)	0.0044 (9)
C15	0.0723 (16)	0.0412 (11)	0.0243 (10)	0.0009 (10)	0.0023 (10)	0.0027 (8)
C16	0.0385 (11)	0.0488 (11)	0.0193 (8)	0.0033 (9)	0.0031 (8)	−0.0040 (8)

### Geometric parameters (Å, °)

**Table:**

Ag1—N1	2.2413 (15)	C5—H5A	0.9300
Ag1—N1^i^	2.2413 (15)	C6—C7	1.431 (3)
Ag1—O2^ii^	2.6585 (17)	C7—C8	1.363 (3)
Ag1—O2^iii^	2.6585 (17)	C7—C16	1.521 (2)
N1—C9	1.320 (2)	C8—C9	1.415 (2)
N1—C1	1.377 (2)	C8—H8A	0.9300
O1—C16	1.280 (2)	C9—C10	1.487 (3)
O1—H1O	0.8200	C10—C15	1.392 (3)
O2—C16	1.223 (3)	C10—C11	1.395 (3)
O2—Ag1^iii^	2.6585 (17)	C11—C12	1.388 (3)
C1—C2	1.414 (3)	C11—H11A	0.9300
C1—C6	1.421 (3)	C12—C13	1.376 (4)
C2—C3	1.360 (3)	C12—H12A	0.9300
C2—H2A	0.9300	C13—C14	1.382 (4)
C3—C4	1.403 (4)	C13—H13A	0.9300
C3—H3A	0.9300	C14—C15	1.380 (3)
C4—C5	1.359 (3)	C14—H14A	0.9300
C4—H4A	0.9300	C15—H15A	0.9300
C5—C6	1.421 (3)		
			
N1—Ag1—N1^i^	177.38 (8)	C8—C7—C16	118.96 (17)
N1—Ag1—O2^ii^	97.54 (6)	C6—C7—C16	123.08 (16)
N1^i^—Ag1—O2^ii^	82.16 (6)	C7—C8—C9	121.59 (18)
N1—Ag1—O2^iii^	82.16 (6)	C7—C8—H8A	119.2
N1^i^—Ag1—O2^iii^	97.54 (6)	C9—C8—H8A	119.2
O2^ii^—Ag1—O2^iii^	167.15 (8)	N1—C9—C8	121.72 (17)
C9—N1—C1	118.51 (16)	N1—C9—C10	118.39 (15)
C9—N1—Ag1	120.76 (12)	C8—C9—C10	119.90 (16)
C1—N1—Ag1	118.86 (11)	C15—C10—C11	118.69 (18)
C16—O1—H1O	109.5	C15—C10—C9	120.59 (18)
C16—O2—Ag1^iii^	137.88 (16)	C11—C10—C9	120.70 (17)
N1—C1—C2	117.98 (17)	C12—C11—C10	120.3 (2)
N1—C1—C6	122.71 (17)	C12—C11—H11A	119.8
C2—C1—C6	119.30 (17)	C10—C11—H11A	119.8
C3—C2—C1	120.9 (2)	C13—C12—C11	120.3 (2)
C3—C2—H2A	119.6	C13—C12—H12A	119.9
C1—C2—H2A	119.6	C11—C12—H12A	119.9
C2—C3—C4	119.9 (2)	C12—C13—C14	119.8 (2)
C2—C3—H3A	120.0	C12—C13—H13A	120.1
C4—C3—H3A	120.0	C14—C13—H13A	120.1
C5—C4—C3	121.1 (2)	C15—C14—C13	120.5 (2)
C5—C4—H4A	119.4	C15—C14—H14A	119.8
C3—C4—H4A	119.4	C13—C14—H14A	119.8
C4—C5—C6	120.6 (2)	C14—C15—C10	120.5 (2)
C4—C5—H5A	119.7	C14—C15—H15A	119.8
C6—C5—H5A	119.7	C10—C15—H15A	119.8
C5—C6—C1	118.14 (18)	O2—C16—O1	125.35 (19)
C5—C6—C7	124.57 (18)	O2—C16—C7	120.26 (18)
C1—C6—C7	117.24 (16)	O1—C16—C7	114.37 (17)
C8—C7—C6	117.95 (17)		

Symmetry codes: (i) −*x*+1, *y*, −*z*−1/2; (ii) *x*−1/2, −*y*+1/2, *z*−1/2; (iii) −*x*+3/2, −*y*+1/2, −*z*.

### Hydrogen-bond geometry (Å, °)

**Table:**

*D*—H···*A*	*D*—H	H···*A*	*D*···*A*	*D*—H···*A*
O1—H1O···O1^iv^	0.82	1.68	2.470 (3)	162

Symmetry codes: (iv) −*x*+1, *y*, −*z*+1/2.
