# Poly[bis­(μ-hemihydrogen 2-phenyl­quinoline-4-carboxyl­ato-κ^2^ *N*,*O*)silver(I)]. Corrigendum

**DOI:** 10.1107/S160053680900628X

**Published:** 2009-02-25

**Authors:** Xiutang Zhang, Peihai Wei, Bin Li

**Affiliations:** a Advanced Material Institute of Research, Department of Chemistry and Chemical Engineering, Shandong Institute of Education, Jinan, 250013, People’s Republic of China; b College of Chemistry and Chemical Engineering, Liaocheng University, Liaocheng, 252059, People’s Republic of China; c Department of Chemistry and Chemical Engineering, Shandong Institute of Education, Jinan 250013, People’s Republic of China

## Abstract

The affiliation of one of the authors in the paper by Zhang, Wei & Li [*Acta Cryst.* (2009), E**65**, m223] is corrected.

## Abstract 2

Corrigendum to *Acta Cryst.* (2009), E**65**, m223.

In the paper by Zhang, Wei & Li [*Acta Cryst.* (2009), E**65**, m223], the affiliation of the second author was given incorrectly. The correct address is given here.

